# The LDL-C/ApoB ratio predicts cardiovascular and all-cause mortality in the general population

**DOI:** 10.1186/s12944-023-01869-1

**Published:** 2023-07-21

**Authors:** Li Xiao, Kerui Zhang, Fang Wang, Min Wang, Qingxia Huang, Chenchen Wei, Zhongshan Gou

**Affiliations:** Center for Cardiovascular Disease, The Affiliated Suzhou Hospital of Nanjing Medical University, Suzhou Municipal Hospital, Gusu School, Nanjing Medical University, Guangji Road, Jiangsu 215002 Suzhou, China

**Keywords:** LDL-C, ApoB, All-cause mortality, Cardiovascular mortality, NHANES

## Abstract

**Background:**

Generally, low-density lipoprotein (LDL) particle size can be inferred from the LDL cholesterol concentration to total apolipoprotein B concentration ratio (LDL-C/ApoB ratio, hereinafter called LAR), which is a good predictor of cardiovascular disease. However, the predictive ability of LAR for mortality risk in the general population is still unclear. This study aimed to explore the association between LAR and cardiovascular as well as all-cause mortality among American adults.

**Methods:**

The present study was a secondary analysis of existing data from the National Health and Nutrition Examination Survey (NHANES). The final analysis included 12,440 participants from 2005 to 2014. Survival differences between groups were visualized using Kaplan‒Meier curves and the log-rank test. The association of LAR with cardiovascular and all-cause mortality was evaluated using multivariate Cox regression and restricted cubic spline analysis. Age, sex, coronary artery disease, diabetes, lipid-lowering medication use and hypertriglyceridemia were analyzed in subgroup analyses.

**Results:**

The median age in the study cohort was 46.0 years [interquartile range (IQR): 31.0–62.0], and 6,034 (48.5%) participants were male. During the follow-up period, there were 872 (7.0%) all-cause deaths and 150 (1.2%) cardiovascular deaths. Compared with individuals without cardiovascular events, those who experienced cardiovascular deaths had a lower LAR (1.13 *vs.* 1.25) (*P* < 0.001). The adjusted Cox regression model indicated that lower LAR was an independent risk factor for both cardiovascular [hazard ratio (HR) = 0.304, 95% confidence interval (CI): 0.114–0.812] and all-cause mortality (HR = 0.408, 95% CI: 0.270–0.617). Moreover, a significant age interaction was observed (*P* for interaction < 0.05), and there was a strong association between LAR and mortality among participants over 65 years of age. Further analysis showed an inverse association between LAR and both cardiovascular and all-cause mortality.

**Conclusions:**

LAR can independently predict cardiovascular and all-cause mortality in the general population.

## Background

Abnormal lipid metabolism has been closely linked to cardiovascular disease (CVD), which is currently the leading cause of death worldwide [1, 2]. It is widely known that an increase in low-density lipoprotein cholesterol (LDL-C) levels is a significant risk factor for the development and progression of atherosclerosis and coronary artery disease (CAD), making LDL-C the primary target for cholesterol-lowering therapy [3–5]. However, in clinical practice, a considerable proportion of patients with normal LDL-C concentrations still experience atherosclerosis, which leads to careful consideration of LDL-C concentrations as a sole indicator [6]. Sachdeva et al. [7] followed up with CAD patients who had been hospitalized continuously for 6 years and found that nearly half of them had relatively normal LDL-C concentrations of < 100 mg/dL. An investigation by Superko and Gadesam [8] demonstrated that particle size was another important index that affected the atherogenicity of LDL in addition to the serum concentration. When serum LDL-C concentrations were fixed, it was observed that the smaller the particle size was, the higher the risk of long-term ischemic heart disease [9]. With age, LDL subfraction profile would shift more and more from a healthy “pattern A” (major LDL peak > 255 A) to “pattern B” (representing small, dense LDL (sdLDL) particles) [10]. Several prospective cohort studies have shown a significant correlation between a high proportion of sdLDL particles and an increased risk of CVD [11–13].

Nuclear magnetic resonance spectroscopy is a valid technology for the measuring of lipoprotein profile, and the results are not easily compromised by lipoprotein composition. However, stringent assay conditions and expensive instruments make it difficult to popularize [14, 15]. Ion mobility analysis and vertical auto profile are also feasible alternative methods [16]. Given the complexity of other traditional testing technologies, such as polyacrylamide gel electrophoresis and density ultracentrifugation, the LDL-C concentration to total apolipoprotein B (ApoB) concentration ratio (LDL-C/ApoB ratio, hereinafter called LAR) could be a more accessible and substantially cheaper tool to estimate LDL particle size [17]. ApoB is the main protein component of LDL and plays a critical role in the transport and clearance of cholesterol in the vascular wall. When LAR is below 1.2 (LDL particle size is approximately 25.5 nm or less), proatherogenic sdLDL is abundantly present [18].

Previous studies on the role of LAR in diseases have mainly focused on evaluating atherosclerotic lipid profiles and predicting the risk of suffering from CVD [19–21]. A recent prospective cohort study [22] found that LAR can predict adverse cardiovascular outcomes among individuals with established atherosclerosis. Similarly, the LURIC Study [23] also showed that LDLapoB/LDL-C ratios were independently associated with cardiovascular mortality. However, to our knowledge, there are currently no studies that focus on LAR to predict mortality events in the general population. Accordingly, our study aimed to investigate the association between LAR and mortality events, i.e., its potential predictive ability for prognosis among general adults.

## Methods

### Study population

**Fig. 1:**
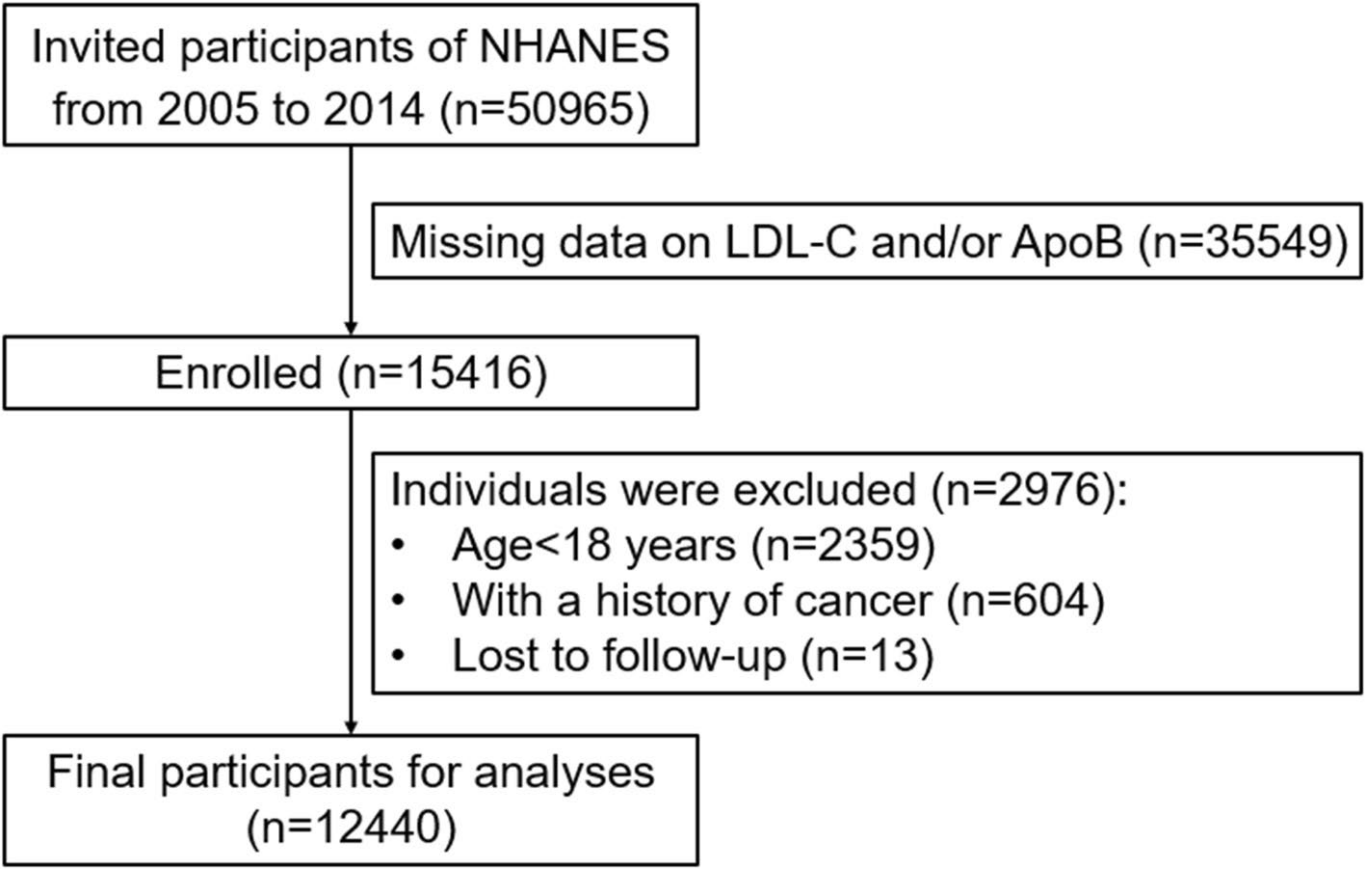
Flow diagram of study selection. NHANES, National Health and Nutrition Examination Survey; LDL-C, low-density lipoprotein cholesterol; ApoB, apolipoprotein B

The study population was selected from the official website of the National Health and Nutrition Examination Survey (NHANES), which is a sequence of multistage surveys that represent the noninstitutionalized populace of the United States. The detailed protocol is provided in NHANES procedure manual [24]. From 2005 to 2014, a total of 50,965 participants were included in the health survey. All subjects with available data on LDL-C and ApoB were enrolled in this study (*n* = 15,416). In this study, 2,359 cases aged < 18 years, 604 cases with cancer and 13 cases who were lost to follow-up were excluded (Fig. 1). The remaining subjects (*n* = 12,440) were successfully included for further analysis. This study passed the ethical review process, and all participants provided written informed consent.

### Assessment of exposure

All participants were instructed to provide fasting blood samples according to the standard protocol. The determination of total cholesterol (TC) and triglyceride (TG) concentrations was based on enzymatic methods. Serum high-density lipoprotein cholesterol (HDL-C) concentrations were determined by either the heparin-manganese precipitation method or direct immunoassay, while ApoB concentrations were measured by immunonephelometry [25]. LDL-C was estimated via the Friedewald formula. The assays were conducted on a Hitachi 704/717 Analyzer or Roche Modular P Chemistry Analyzer [26].

### Assessment of covariates

Participants were interviewed either in person or via computer-assisted personal interview (CAPI). All respondents completed a questionnaire providing demographic and health information. Demographic information, such as date of birth, sex, race, health information (including medical history and smoking and drinking habits), and medications at the time of enrollment, was collected. Information on a history of the following medical conditions was collected according to self-reported data: hypertension, diabetes mellitus, CAD and stroke. Smokers were defined as those who had smoked for more than 6 months or over 100 cigarettes accumulatively, and drinkers were defined as those who had at least 12 drinks in the past 12 months [27]. Participants with systolic blood pressure ≥ 140 mmHg and/or diastolic blood pressure ≥ 90 mmHg were categorized as having hypertension, and individuals with a fasting blood glucose concentration ≥ 7 mmol/L or glycated hemoglobin (HbA1c) concentration ≥ 6.5% were considered to have diabetes [25].

### Outcome ascertainment

The etiologies of mortality were classified according to the tenth revision of the International Classification of Diseases (ICD-10) [28]. The study endpoint was all-cause and cardiovascular mortality, defined as any cardiovascular disease-related death (ICD-10 codes I00-I99). Data on deaths were obtained by cross-linking the NHANES datasets to the National Death Index [29]. Each participant’s follow-up duration commenced on the date of their survey participation and ended on either the date of their death or the end of the follow-up period (December 31, 2015).

### Statistical analysis

The Shapiro‒Wilk test was conducted to evaluate the normality of the distribution of continuous variables. Nonnormally distributed variables were presented as medians (interquartile ranges, IQRs), while categorical variables were presented as proportions. The Mann‒Whitney U test or χ^2^ test was utilized for group comparisons. Kaplan‒Meier curves and log-rank tests were used to compare survival differences between groups. The Cox proportional hazards model was used to examine the independent association between LAR and all-cause and cardiovascular mortality. Three risk models were created: Model 1 was a crude model without adjustment for confounders. Age, sex, race, alcohol consumption, and smoking status were included as covariates in Model 2. Model 3 included all covariates in Model 2, as well as other conventional cardiovascular risk factors such as hypertension, diabetes, CAD, serum TG levels, and lipid-lowering medication use.

For subgroup analysis, the fully adjusted models were stratified by age, sex, CAD, diabetes, use of lipid-lowering medication and hypertriglyceridemia, and interactions were assessed. Then, we also evaluated the overall effect and linear trend between LAR and mortality risk by restricted cubic spline models. We accounted for the complex survey design and the probability weights were used as recommended by the NCHS in our analysis [30, 31]. Multiple imputation was used to replace missing values. Statistical analysis was performed using R version 3.5.3, with two-tailed tests used for all analyses. A *P* value of less than 0.05 was considered statistically significant.

## Results

### Baseline characteristics

Among 12,440 participants, the overall median age was 46.0 years (IQR 31.0–62.0), and 6,034 (48.5%) were male. The median (IQR) LAR percentile was 1.24 (1.12–1.35), and the percentile distribution ranged from 0.92 to 1.51 (5-95th percentile) and from 0.74 to 1.63 (1-99th percentile). During a median follow-up of 68 months, 872 (7.0%) all-cause deaths occurred, 150 (17.2%) of which were CVD-related deaths. According to whether cardiovascular deaths occurred, participants were divided into two groups. Table 1 provides a detailed description of the baseline characteristics of the two groups.

**Table 1:** Demographic and baseline characteristics of participants with or without cardiovascular deaths

	No cardiovascular death (*n* = 12,290)	Cardiovascular death (*n* = 150)	*P* value
Age (years)	46.0 (31.0, 62.0)	75.0 (65.3, 80.0)	< 0.001
Age group			
< 65 years	9779 (79.6%)	33 (22.0%)	< 0.001
≥ 65 years	2511 (20.4%)	117 (78.0%)	
Gender			
Male	5936 (48.3%)	98 (65.3%)	< 0.001
Female	6354 (51.7%)	52 (34.7%)	
Race			
Mexican American	2081 (16.9%)	15 (10.0%)	< 0.001
Non-Hispanic white	5299 (43.1%)	93 (62.0%)	
Non-Hispanic black	2596 (21.1%)	29 (19.3%)	
Other races	2314 (18.8%)	13 (8.7%)	
Drinking	8774 (71.4%)	107 (71.3%)	0.988
Smoking	5402 (44.0%)	89 (59.3%)	< 0.001
Hypertension	4586 (37.3%)	110 (73.3%)	< 0.001
Diabetes	1879 (15.3%)	48 (32.0%)	< 0.001
CAD	1288 (10.5%)	35 (23.3%)	< 0.001
Stroke	681 (5.5%)	19 (12.5%)	0.011
Lipid-lowering drugs	2588 (21.1%)	67 (44.7%)	< 0.001
TC (mmol/L)	4.86 (4.19, 5.59)	4.82 (4.09, 5.61)	0.563
TG (mmol/L)	1.15 (0.81, 1.69)	1.33 (1.02, 2.04)	< 0.001
HDL-C (mmol/L)	1.34 (1.11, 1.63)	1.27 (1.09, 1.58)	0.121
LDL-C (mmol/L)	2.85 (2.28, 3.50)	2.76 (2.07, 3.47)	0.191
ApoB (mg/dL)	89.0 (73.0, 107.0)	96.0 (75.0, 113.5)	0.049
LAR	1.25 (1.12, 1.35)	1.13 (1.01, 1.24)	< 0.001

Data were presented as median (IQR) or N (%). *CAD* Coronary artery disease, *TC *Total cholesterol, *TG* Triglycerides, *HDL-C* High-density lipoprotein cholesterol, *LDL-C* Low-density lipoprotein cholesterol, *ApoB* Apolipoprotein B, *LAR* The LDL cholesterol concentration to total apolipoprotein B concentration ratio

Compared with the participants without cardiovascular deaths, those who experienced cardiovascular deaths were older, predominantly male, and had a greater proportion of smokers (*P* < 0.05). The occurrence of any coexisting cardio-cerebrovascular diseases, including hypertension, diabetes, CAD and stroke, was higher in the population with cardiovascular deaths, as was the use of lipid-lowering medication (all *P* values < 0.05). Moreover, serum TG concentrations were significantly higher in individuals who experienced cardiovascular death (*P* < 0.05). Importantly, LAR displayed obvious downward trends in participants who experienced cardiovascular death (*P* < 0.001).

### Association of LAR with cardiovascular mortality

**Fig. 2:**
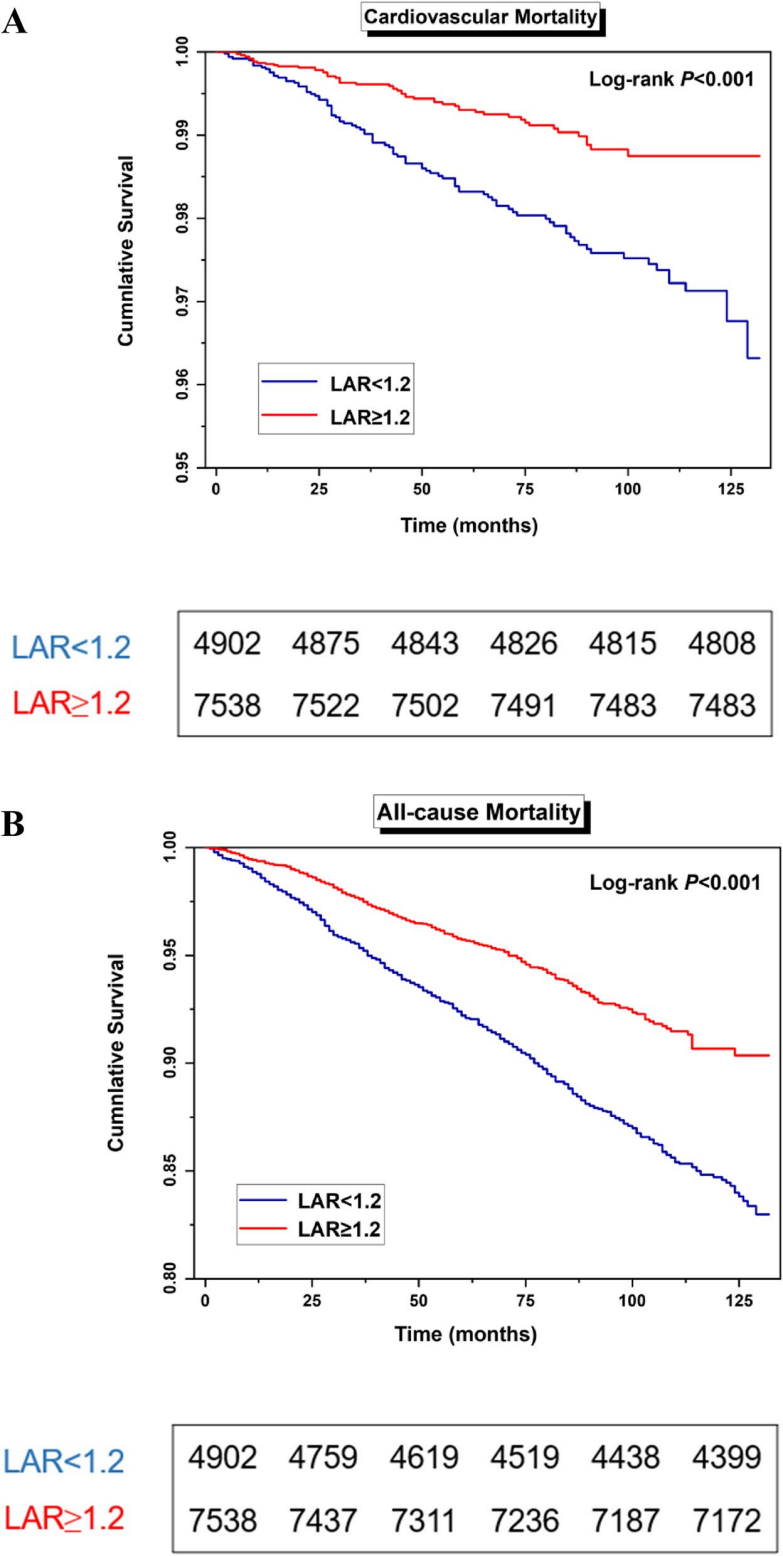
**A** Kaplan-Meier survival analysis for cardiovascular mortality (LAR <1.2 *vs.* ≥1.2). **B** Kaplan-Meier survival analysis for all-cause mortality (LAR <1.2 *vs.* ≥1.2)

**Fig. 3:**
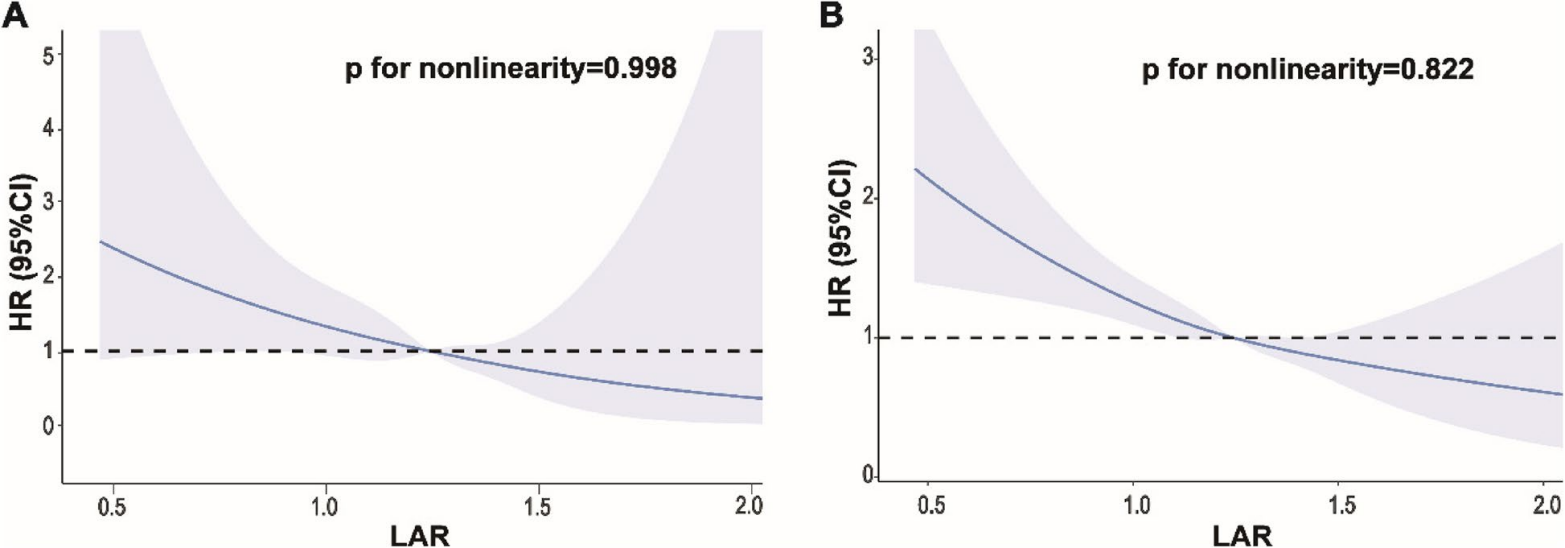
Restricted cubic spline plots of the association between LAR with cardiovascular mortality (**A**) and all-cause mortality (**B**) in the general population. Analysis was adjusted for age, sex, race, drinking, smoking, hypertension, diabetes, coronary artery disease, serum triglyceride level and lipid-lowering drugs. HR, hazard ratio

Participants were categorized into two groups based on whether their baseline LAR was above or below 1.2. Kaplan‒Meier survival curves showed a notable difference in cardiovascular death risk between the groups (*P* for log-rank test < 0.001, Fig. 2A). After adjusting for possible confounding variables, the final Cox proportional hazards regression model demonstrated a statistically significant association between a decline in LAR and an elevated risk of cardiovascular mortality (HR = 0.304, 95% CI: 0.114–0.812, Table 2). The restricted cubic spline model showed that there was a linear association of LAR with cardiovascular mortality (*P* for nonlinearity = 0.998, Fig. 3A).

**Table 2:** Multivariate Cox regression analyses of LAR association with mortality

	Model 1 h (95% CI)	*P* value	Model 2 h (95% CI)	*P* value	Model 3 h (95% CI)	*P* value
Cardiovascular mortality						
LAR	0.075 (0.035, 0.163)	< 0.001	0.222 (0.096, 0.510)	< 0.001	0.304 (0.114, 0.812)	0.018
LAR group						
Group 1 (≥ 1.2)	Reference		Reference		Reference	
Group 2 (< 1.2)	2.320 (1.661, 3.240)	< 0.001	1.506 (1.074, 2.113)	0.018	1.299 (0.900, 1.873)	0.162
All-cause mortality						
LAR	0.151 (0.108, 0.211)	< 0.001	0.417 (0.293, 0.594)	< 0.001	0.408 (0.270, 0.617)	< 0.001
LAR						
Group 1 (≥ 1.2)	Reference		Reference		Reference	
Group 2 (< 1.2)	1.819 (1.589, 2.083)	< 0.001	1.258 (1.097, 1.443)	0.001	1.223 (1.054, 1.419)	0.008

*h *Hazard ratio, *CI *Confidence interval

Model 1: non-adjusted

Model 2: adjusted for age, sex, race, drinking, and smoking

Model 3: adjusted for age, sex, race, drinking, smoking, hypertension, diabetes, coronary artery disease, serum triglyceride level, and lipid-lowering drugs

### Association of LAR with all-cause mortality

The Kaplan–Meier curves indicated a significant association between different LAR groups and overall survival rate (*P* < 0.001, Fig. 2B). LAR demonstrated a clear association with all-cause mortality in the Cox model without adjustment (HR = 0.151, 95% CI: 0.108–0.211). To exclude the influence of the confounders, potential confounders were incorporated as adjustment factors into multivariate regression. LAR remained an independent predictor of all-cause mortality (HR = 0.408, 95% CI: 0.270–0.617). The risk of all-cause mortality in the low ratio group (LAR < 1.2) was approximately 1.22 times greater than that in the high ratio group (HR = 1.223, 95% CI: 1.054–1.419, Table 2). As shown by restricted cubic spline analyses (Fig. 3B), HRs for all-cause mortality gradually increased with decreasing LAR (*P* for nonlinearity = 0.822).

### Subgroup analysis

**Fig. 4:**
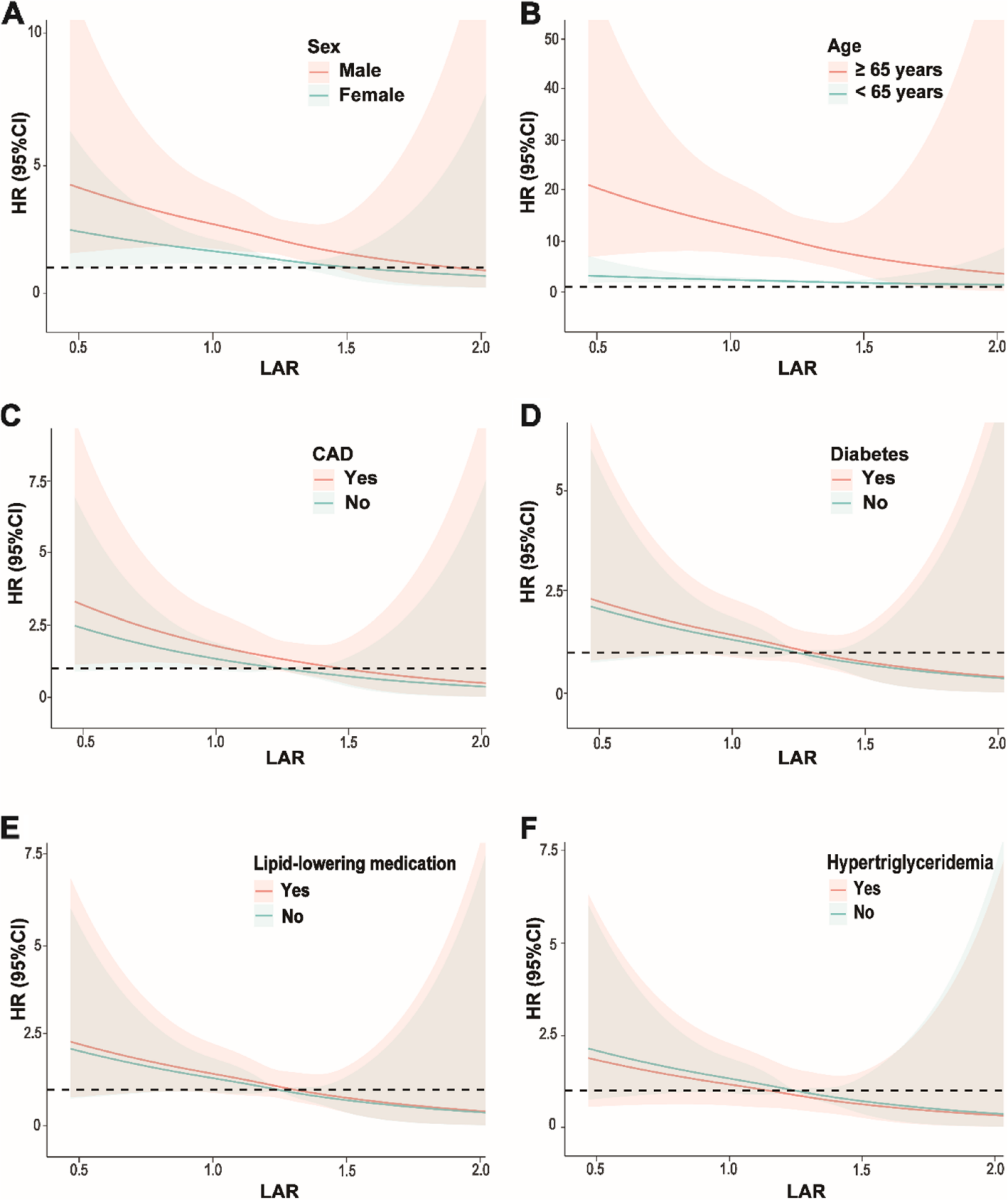
Subgroup analysis of restricted cubic spline plots for the association between LAR and cardiovascular mortality by sex (**A**), age (**B**), CAD (**C**), diabetes (**D**), lipid-lowering medication (**E**) and hypertriglyceridemia (**F**)

**Fig. 5:**
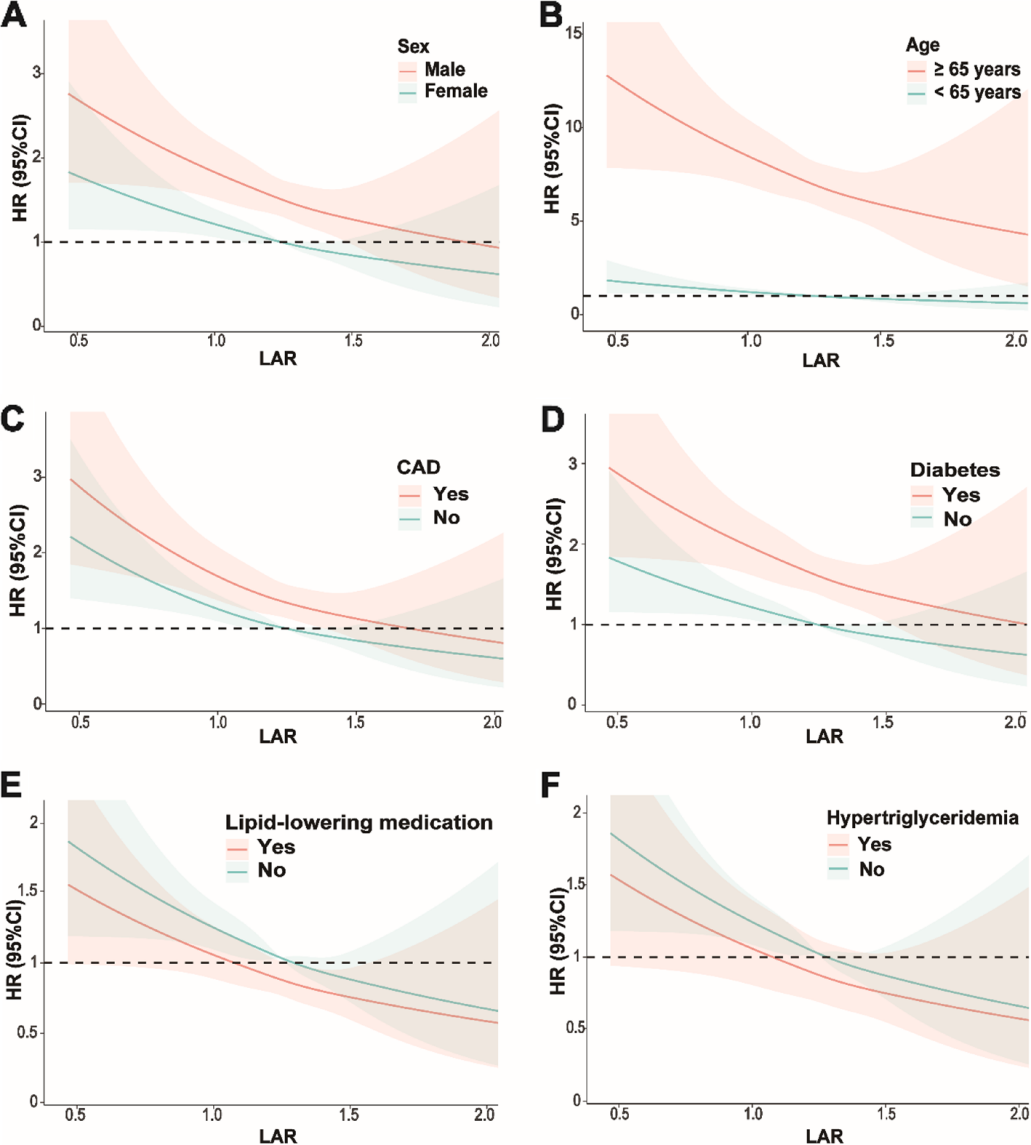
Subgroup analysis of restricted cubic spline plots for the association between LAR and all-cause mortality by sex (**A**), age (**B**), CAD (**C**), diabetes (**D**), lipid-lowering medication (**E**) and hypertriglyceridemia (**F**)

To further investigate the association of LAR with cardiovascular and all-cause mortality, stratified analyses by age, sex, CAD, diabetes, use of lipid-lowering medication and hypertriglyceridemia were performed (Table 3). Among participants of different sexes or with and without CAD, diabetes, lipid-lowering medication and hypertriglyceridemia, there was no significant difference in the association of LAR with mortality (*P* for interaction > 0.05). In addition, there was a significant interaction between LAR and age (*P* for interaction < 0.001) in terms of mortality, and the association of LAR with cardiovascular and all-cause mortality was more pronounced in participants over 65 years of age (*P* < 0.05). When using restricted cubic spline plots to depict LAR as a continuous variable, a linear correlation was observed between LAR and both cardiovascular and all-cause mortality rates in subgroups stratified by age, sex, CAD, diabetes, lipid-lowering medication use and hypertriglyceridemia (Figs. 4 and 5).

**Table 3:** Subgroup analysis for the association between LAR and mortality

	Cardiovascular mortality				All-cause mortality				
	N (%)	LAR HR (95% CI)	*P* value	p-int	N (%)		LAR HR (95% CI)	*P* value	p-int
Age				0.044					< 0.001
<65 years	33 (0.3%)	0.496 (0.173, 1.425)	0.193		255 (2.6%)	0.686 (0.436, 1.080)		0.103	
≥ 65 years	117 (4.5%)	0.071 (0.013, 0.395)	0.003		617 (23.5%)	0.164 (0.083, 0.325)		< 0.001	
Gender				0.487					0.066
Male	98 (1.6%)	0.209 (0.050, 0.877)	0.032		516 (8.6%)	0.282 (0.160, 0.498)		< 0.001	
Female	52 (0.8%)	0.382 (0.118, 1.238)	0.109		356 (5.6%)	0.545 (0.325, 0.912)		0.021	
CAD				0.978					0.636
Yes	35 (2.6%)	0.298 (0.055, 1.633)	0.163		190 (14.4%)	0.476 (0.223, 1.013)		0.054	
No	115 (1.0%)	0.306 (0.102, 0.922)	0.035		682 (6.1%)	0.389 (0.246, 0.615)		< 0.001	
Diabetes				0.124					0.258
Yes	48 (2.5%)	0.749 (0.165, 3.412)	0.709		312 (16.2%)	0.529 (0.287, 0.972)		0.040	
No	102 (1.0%)	0.187 (0.059, 0.593)	0.004		560 (5.3%)	0.346 (0.209, 0.572)		< 0.001	
Lipid-lowering medication				0.843					0.116
Yes	67 (2.5%)	0.275 (0.068, 1.117)	0.071		316 (11.9%)	0.602 (0.320, 1.133)		0.116	
No	83 (0.8%)	0.327 (0.097, 1.095)	0.070		556 (5.7%)	0.331 (0.204, 0.538)		< 0.001	
Hypertriglyceridemia				0.064					0.355
Yes	50 (1.6%)	0.814 (0.185, 3.586)	0.785		246 (8.0%)	0.545 (0.286, 1.039)		0.065	
No	100 (1.1%)	0.143 (0.044, 0.467)	0.001		626 (6.7%)	0.376 (0.231, 0.611)		< 0.001	

* h *Hazard ratio, *CI *Confidence interval, *p-int *p for interaction. Hypertriglyceridemia was defined as serum TG level > 1.7 mmol/L. Adjusted for age, sex, race, drinking, smoking, hypertension, diabetes, coronary artery disease, serum triglyceride level, and lipid-lowering drugs as required

## Discussion

LAR is commonly used as an indirect marker of LDL particle size and is associated with the development of atherosclerosis and adverse cardiovascular outcomes. This is the first study to evaluate the long-term prognostic efficacy of LAR among general adults. This study demonstrated that a decreased LAR was linked to higher all-cause and cardiovascular mortality in the general population, irrespective of other conventional risk factors. In the population studied, there was no significant difference in the association of LAR with the risk of mortality among subjects of different genders, history of CAD, diabetes and hypertriglyceridemia, and a history of lipid-lowering medication. Further analysis suggested that there was a negative linear association between LAR and mortality.

The majority of international guidelines agree that reducing circulating LDL concentrations is one of the main goals for the prevention of atherosclerotic cardiovascular disease [32–34]. LDL is a heterogeneous population of particles with relatively discrete rather than continuously variable size and density [35]. Specifically, LDL is subdivided into seven subcategories (LDL-1 to LDL-7) in nondenaturing gradient gel electrophoresis, ranging from largest and most buoyant to smallest and densest [36]. LDL-3 to 7 is also referred to as sdLDL. A previous study [13] found that sdLDL was the most atherogenic parameter, even more than LDL, which is related to its biochemical properties. The smaller size makes it easier for LDL particles to penetrate the arterial wall and escape receptor-mediated uptake, leading to increased atherogenic risk [37, 38]. Moreover, its longer operational half-life predisposes atherogenic modification [39]. High concentrations of sdLDL have been unequivocally demonstrated as a risk factor for CVD [38, 40, 41]. A study among older men with stable CAD by Sakai et al. [42] also indicated that sdLDL-C was a superior biomarker to LDL-C to predict future cardiovascular events. ApoB-100, a 550 kDa glycoprotein synthesized in the liver, is the major apolipoprotein contained in LDL particles and serves as the binding domain for the LDL receptor [43]. It has been substantiated that ApoB is comparable to non-HDL cholesterol in predicting future CAD risk, while it may be even superior to non-HDL cholesterol in predicting cardiovascular events [44–46]. Studies [47, 48] have suggested that each LDL particle has a single ApoB molecule and more than 90% of all ApoB is found in LDL; of course, a minor fraction of ApoB is also carried on other atherogenic particles (e.g., very-low density lipoproteins and intermediate density lipoproteins); thus, plasma ApoB broadly reflects the quantity of LDL particles. Hence, the significance of LAR has been discussed, as it can indicate the approximate size of LDL particles and assess the relative predominance of sdLDL. In individuals with low ratio values, sdLDL has a higher predominance in LDL particles in plasma.

The measurement of LAR has gained increased attention in recent years. Multiple studies [49–52] have indicated that LAR is associated with cardiovascular diseases and the development of cardiovascular diseases later in life. Drexel et al. [22] recently reported that LAR can predict major adverse cardiovascular events in patients with preexisting atherosclerotic cardiovascular diseases. However, the enrolled population of the study was relatively small and limited. Therefore, the present study was conducted in the general population. The sample had wide coverage, and the study population was nationally representative. The results indicated that this ratio was able to predict cardiovascular mortality not only among patients with CAD but also in the general population. LAR of 1.2 serves as a critical value for the classification of sdLDL and large, buoyant LDL [18, 53]. When the ratio is below 1.2, there is a substantial increase in the risk of all-cause mortality.

TGs are considered to be the most powerful determinant of LDL particle size [54]. Most plasma triglycerides are found in lipoproteins that are rich in triglycerides (TRLs), including chylomicrons and very low-density lipoprotein (VLDL), and their metabolism is inextricably linked to cholesterol metabolism [55]. Tani et al. conducted a cross-sectional study [56] and found that the estimated LDL particle size (based on LAR) showed significant inverse correlations with most markers related to TG-rich lipoproteins, including TG concentration, especially among CAD patients with diabetes mellitus. Experimental studies suggest that sdLDL, stimulated by hypertriglyceridemia, may be more susceptible to oxidative modification and concomitant dysfunctional HDL and may adversely affect atherosclerosis [56–58]. Considering that abnormal TG metabolism seems to lead to a reduction in LDL particle size, hypertriglyceridemia was included as a confounding factor in the multivariate regression analysis to eliminate its effect. The effective predictive ability of LAR for all-cause and cardiovascular mortality persisted.

Statins are the most commonly used drugs in the clinic to control hyperlipidemia and prevent cardiovascular disease. However, a recent study [59] have suggested that statins likely play a more complex role than simply lowering serum lipid concentrations. The reported benefits of statin therapy might be overestimated and exaggerated, and their clinical efficacy has also been questioned by some scholars [60]. Statins may have implications for many other biological pathways, and they have been reported to have effects on LDL particle size [61, 62]. In a multicenter randomized study [59], patients with familial combined hyperlipidemia were treated with two statins. However, different responses were observed: atorvastatin increased the average LDL particle size, while pravastatin reduced the particle size. Therefore, in our study, multivariate analyses were utilized to account for the use of lipid-lowering drugs and demonstrated that the significance of LAR as a predictor of mortality persisted in our particular context. Furthermore, stratified analysis and the test for interaction showed that the association between LAR and mortality did not change with the use of lipid-lowering medications. Given that different drugs affect LDL particle size differently, further detailed research is needed to clarify the influence of different lipid-lowering drugs on the relationship between LAR and mortality.

### Comparisons with other studies and what does the current work add to the existing knowledge

Previous studies [19–21] have mainly focused on the association between LAR and the development of cardiovascular disease. To our knowledge, only the study by Drexel et al. [22] and Silbernagel et al. [23] have evaluated the prognostic value of LAR for death. In contrast, their studies were only conducted in CAD patients, while we expanded firstly the scope to the general population. This further demonstrated the importance of this indicator. Our findings might provide a theoretical basis for the predictive value of LAR in the general population, which is worth further promoting and utilization in clinical practice.

### Study strengths and limitations

The present study had several key points and strengths. First, the sample was large and had extensive coverage. The study population was largely representative of the general US adult population. Second, our retrospective cohort study presented firm evidence of the prognostic efficiency of LAR for both cardiovascular and all-cause mortality in the general population. Prior to this study, similar studies were only conducted among patients with CAD. Finally, we used a restricted cubic spline model to visually demonstrate the relationship between LAR and the long-term risk of mortality for the first time.

There were also several potential limitations. Principally, LAR was a rough estimate of LDL particle size rather than a direct measurement tool. And measurement of LDL-C, ApoB and covariates was limited to the baseline in this study, but they might have changed during follow-up. The ratio could not represent the long-term levels in the population. Second, as medication intake and physical activity could not be completely followed up and systematically analyzed, the possible effects of lipid-regulating agents and physical activity on the results cannot be excluded. Moreover, the Friedewald formula is still the main method for the assessment of serum LDL-C concentrations because of its low cost and convenience. With the improved accuracy of the direct method of LDL-C measurement, it can be expected that it will be substituted for the Friedewald formula. The data were derived from residents of the United States; therefore, future studies can be conducted in other countries to determine whether these results are valid worldwide.

## Conclusions

In conclusion, the current study provided evidence of an independent association of LAR, serving as an estimation indicator of LDL particle size, with both cardiovascular and all-cause mortality. In addition to serum LDL concentrations, LAR may also function as another valuable indicator of poor prognosis, which may be relevant for residual cardiovascular risk.

## Data Availability

The datasets used during the current study are available from the corresponding author on reasonable request.

## Abbreviations

ApoB: Apolipoprotein B
CAD: Coronary artery disease
CVD: Cardiovascular disease
HDL-C: High-density lipoprotein cholesterol
LAR: The LDL cholesterol concentration to apolipoprotein B concentration ratio
LDL-C: Low-density lipoprotein cholesterol
NHANES: National Health and Nutrition Examination Survey
sdLDL: Small dense low-density lipoprotein
TC: Total cholesterol
TG: Triglyceride
